# EUS Shear Wave Measurement for Early Chronic Pancreatitis: A Retrospective Diagnostic Accuracy Study Using the 2019 JPS Criteria

**DOI:** 10.3390/jcm14207349

**Published:** 2025-10-17

**Authors:** Muneo Ikemura, Yusuke Takasaki, Koichi Ito, Akinori Suzuki, Yuka Fukuo, Hironao Okubo, Hiroyuki Isayama

**Affiliations:** 1 Department of Gastroenterology, Graduate School of Medicine, Juntendo University, Tokyo 113-8421, Japan; m-ikemura@juntendo.ac.jp (M.I.); ytakasa@juntendo.ac.jp (Y.T.); kitoh@juntendo.ac.jp (K.I.); suzukia@juntendo.ac.jp (A.S.); 2 Department of Gastroenterology, Juntendo University Nerima Hospital, Tokyo 177-8521, Japan; yfukuo@juntendo.ac.jp (Y.F.); drokubo@juntendo.ac.jp (H.O.); 3 Division of Gastroenterology, Department of Medicine, Faculty of Medicine, Chulalongkorn University, Bangkok 10330, Thailand

**Keywords:** EUS elastography, early chronic pancreatitis, shear wave measurement

## Abstract

**Background/Objectives:** Diagnosis of early chronic pancreatitis (eCP) is important to prevent progression to established CP. Endoscopic ultrasonography (EUS) plays an important role in diagnosis; however, it is still difficult and has drawbacks in its inability to assess progression. EUS shear wave measurement (EUS-SWM) has been established to be a precise method for evaluating stiffness and the degree of fibrosis. This study aimed to evaluate the utility of EUS-SWM in eCP findings. **Method:** This study was retrospective, single center, and identified the 2019 Japan Pancreas Society (JPS) eCP criteria as the reference standard. We evaluated 38 patients who underwent EUS-SWM between April and November 2022. Patients were classified into positive EUS findings based on the JPS criteria for less than 2 (non-eCP finding group) and 2 or more (eCP finding group). The EUS-SWM value was compared between these groups and the relationship was analyzed with EUS findings. **Result:** Eleven of thirty-eight patients had eCP findings. The EUS-SWM value is significantly higher in the eCP finding group (2.39 ± 0.54 vs. 1.84 ± 0.46, *p* = 0.0065), and the EUS-SWM value is positively correlated with the number of positive EUS findings (r = 0.62, *p* < 0.001). The area under the receiver operating characteristic curve for EUS-SWM to distinguish the eCP finding group was 0.78 (0.62–0.95). The cut-off value of 1.87 showed sensitivity and specificity of 90.9% and 63.0%, respectively. **Conclusions:** EUS-SWM values correlate with EUS findings in eCP and may serve as an objective biomarker for disease progression.

## 1. Introduction

According to the systematic review by Xiao et al. [1], nationwide epidemiological surveys in Japan have demonstrated increasing prevalence of chronic pancreatitis (CP) from 28.5/100,000 in 1994 to 52.4/100,000 in 2011 [2,3]. Therefore, it is currently considered one of the most important healthcare problems. Whitecomb et al. [4] proposed a mechanistic definition—”chronic pancreatitis is a pathologic fibro-inflammatory syndrome of the pancreas in individuals with genetic, environmental and/or other risk factors who develop persistent pathologic responses to parenchymal injury or stress.” The diagnostic criteria for eCP were first defined by JPS in 2009 [5]. Diagnosing eCP may delay progression to CP by providing patients with guidance that includes abstaining from alcohol. Although diagnosing eCP is clinically important to prevent progression and complications, it can be difficult due to a lack of sensitive blood, functional biomarkers, and treatment. While eCP has attracted growing interest, its pathophysiological basis and clinical relevance remain incompletely clarified. The gold standard for diagnosis is generally histological findings, but obtaining histological findings is difficult in early chronic pancreatitis. In recent years, the relationship between EUS findings and histopathological findings of CP/eCP has become better understood [6,7]. Diagnosing eCP imaging alone is also challenging, given that morphological changes may not appear on imaging until the later stage of the disease. EUS is useful in the diagnosis of eCP because it can capture subtle changes with high resolution, but the interobserver agreement rate is not satisfactory, and the diagnostic criteria remain controversial [8,9,10]. Therefore, JPS criteria 2009 were revised for easier diagnosis in 2019 [11], and their reliability was evaluated previously [12]. EUS-strain elastography was used to improve the diagnostic performance of CP and was shown to be correlated with the CP stages of Rosemont classification [13]. EUS-SWM can enhance the precision of CP diagnosis, as it enables absolute measurement of pancreatic stiffness [14,15,16]. However, there are few reports of the usefulness of EUS-SWM because it is a relatively novel method in the field of EUS [17]. There is also limited data on whether EUS findings in eCP are associated with pancreatic stiffness [18]. Therefore, the evaluation of the usefulness of EUS-SWM in eCP remains controversial. We hypothesized that EUS-SWM could be used as a quantitative biomarker in eCP. In this study, we measured pancreatic stiffness using EUS-SWM and investigated the relationship between EUS findings and pancreatic stiffness.

## 2. Materials and Methods

### 2.1. Study Design

This single-center, retrospective study was performed in Juntendo Nerima Hospital. This retrospective study was approved by the ethics committee of Nerima Hospital of Juntendo University (No. E22-0385, approval 24 November 2022). The study was conducted in accordance with the ethical standards of the 1964 Declaration of Helsinki and its later amendments, with an opt-out consent process. The primary outcome was to measure the correlation between the EUS-SWM value and the number of EUS findings in eCP according to JPS criteria 2019. According to JPS criteria 2019, the EUS findings of eCP include four findings: hyperechoic foci or stranding, lobularity, hyperechoic main pancreatic duct (MPD) margins, and dilated side branches. EUS procedures were performed by a board-certified fellow of the Japan Gastroenterological Society and the JPS, with experience in over 1000 EUS cases (Y.T.). Two endoscopists (K.I. and A.S.), blinded to clinical information, independently evaluated the presence or absence of EUS findings. EUS images other than the EUS-SWM measurement were anonymized and output for evaluation by the respective raters. When the two findings did not match, the findings were determined with reference to the endoscopic operator’s findings.

EUS-SWM values were compared between two groups: those with fewer than two EUS findings (non-eCP finding group) and those with two or more findings (eCP finding group). The secondary outcome was to measure the diagnostic capability of EUS-SWM for the number of EUS findings of the cut-off points for their diagnoses. The optimal cut-off values for EUS-SWM were determined according to their sensitivity and specificity. We also evaluated the correlation between EUS-SWM and heavy alcohol drinkers. Heavy alcohol drinkers are defined as 60 g (pure ethanol amount) or more per day.

### 2.2. Patients

Patients were those with suspected pancreaticobiliary disease who needed to undergo EUS and those who had EUS-SWM measured were consecutively enrolled between April 2022 and November 2022. The inclusion criteria were as follows: age > 18 years; EUS and EUS-SWM were performed on the same day. The exclusion criteria were malignant pancreatic lesion, severe liver function disorder, and previous pancreatic surgical procedure and/or total gastrectomy.

### 2.3. Endoscopic Ultrasonography Procedure and EUS-SWM

**Figure 1:**
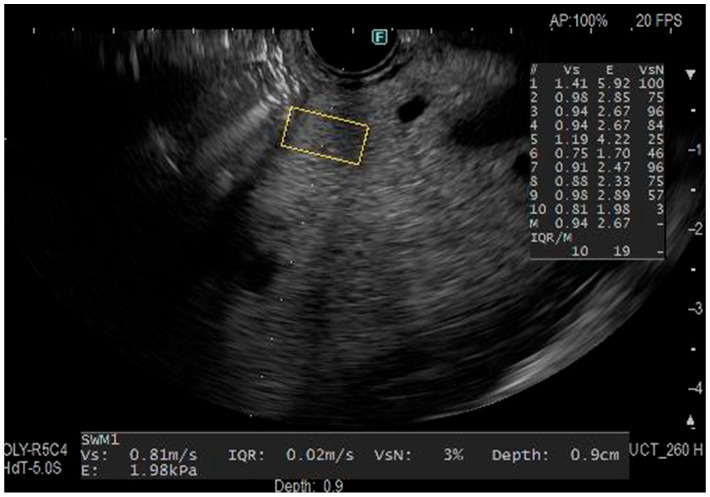
Measurement of EUS-SWM.

Convex-type echo-endoscopes (GF-UCT260; Olympus, Tokyo, Japan) with ultrasound observation systems (ARIETTA 850; Fuji Film healthcare, Ltd., Tokyo, Japan) were used. EUS was performed in the left lateral position under midazolam-induced sedation with monitoring of heart rate, saturation, and blood pressure. The endosonographer recorded EUS images for later evaluation. EUS-SWM was performed after recording the EUS images. The region of interest (ROI) measuring 5 × 10 mm was designated on the pancreatic body parenchyma, with exclusion of vessels, pancreatic duct, and cysts. Ten shear wave velocity (Vs) measurements were obtained using EUS-SWM, and the mean value was calculated. (Figure 1). The percentage of the net amount of effective shear wave velocity (VsN: %) shows which percentage of the measurement value is used in the calculation of Vs. The calculated VsN is used to assess whether the Vs value is reliable.

Representative endoscopic ultrasonography (EUS) image in a patient. The body of the pancreas was measured 10 times from the intragastric manipulation, avoiding the pancreatic ducts and other vessels to the extent possible without applying an up-angle position.

### 2.4. Statistical Analysis

Continuous variables are reported as median with range and mean with standard deviation. Between-group differences were assessed using the chi-squared or Fisher’s exact test for categorical variables and the Wilcoxon rank-sum test for continuous variables. We evaluated the relationship using Spearman’s correlation coefficient. Receiver operating curve (ROC) analysis was conducted to identify the cut-off value of Vs for eCP finings > 2. Area under the ROCs (AUROCs) are presented with 95% confidence intervals (CIs). Since the diagnostic criteria for ECP include subjective components, evaluations were performed by two independent observers, and interobserver agreement was assessed using Cohen’s kappa and Youden’s J statistics, as appropriate. Kappa values were categorized as follows: 0–0.20, slight; 0.21–0.40, fair; 0.41–0.60, moderate; 0.61–0.80, substantial; and 0.81–1.00, almost perfect (Biometrics 1977; 33:159–174). STATA version 13 software (Stata Corp., College Station, TX, USA) was used for all statistical analyses. A *p*-value < 0.05 was considered significant.

## 3. Results

### 3.1. Patient Characteristics

Thirty-eight patients were retrospectively analyzed. The median patient age was 68.5 (range 57.75–75.75) years old. Twenty patients (52.6%) were Male. Ten patients (26.3%) were heavy drinkers, more than 60 g of alcohol per day. Five patients (13.1%) had Diabetes mellitus. In total, 13 patients (34.2%) had pancreatic cysts, 10 patients (26.3%) had chronic pancreatitis, 1 patient (2.6%) had autoimmune pancreatitis, and 2 patients (5.3%) had a history of acute pancreatitis (Table 1).

### 3.2. EUS Findings and EUS-SWM

The overall kappa values (95% CIs) of interobserver reliability (IOR) for each EUS finding were as follows: hyperechoic foci and strands: 0.69 (0.46–0.91), lobularity: 0.48 (−0.12–1.08), hyperechoic MPD margin: 0.43 (0.09–0.78), dilated branch ducts: −0.04 (−0.09–0.01) (Table 2).

Eleven patients (28.9%) showed two or more EUS findings of eCP (eCP finding group), and the remaining patients were classified as the non-eCP finding group. The most common EUS finding of eCP was hyperechoic foci or stranding, which occurred in twenty-one patients (55.3%). Lobularity was in eight (21.6%), hyperechoic MPD margin was in five (13.1%), and dilatated side branch was in eleven (28.9%).

**Figure 2:**
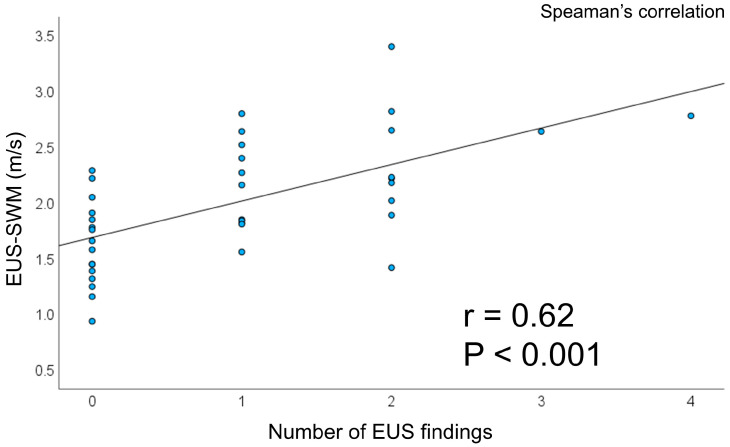
Correlation of EUS-SWM and EUS findings.

Mean EUS-SWM values were significantly higher in heavy drinkers (2.31 ± 0.50 vs. 1.84 ± 0.46, *p* = 0.04). There were 10 (90.9%) males in the eCP finding group and 10 (37.0%) in the non-eCP finding group, respectively. Each mean value of EUS-SWM was 2.39 ± 0.54, 1.84 ± 0.46 (*p* = 0.007), respectively (Table 3). EUS-SWM was significantly higher in the eCP finding group than in the non-eCP finding group. The EUS-SWM value was significantly positively correlated with EUS findings of eCP (r = 0.62, *p* < 0.001) (Figure 2).

Scatter plots of the value of EUS-SWM compared with that of the number of EUS findings. Abbreviations: EUS-SWM, endoscopic ultrasonography—shear wave measurement.

### 3.3. Comparison of Heavy Drinkers and EUS-SWM

The median age of heavy drinkers was 71.5 (range: 56.75–75.75) years old. The median age of non-heavy drinkers was 65.0 (range: 57.25–75.25) years old. Six males (60.0%) were heavy drinkers and fifteen (50.0%) were non-heavy drinkers, respectively. Seven CP patients (70.0%) were heavy alcohol drinkers, and three (11.1%) were non-alcohol drinkers. Five eCP finding group patients (50%) heavy alcohol drinkers, and six (21.4%) were non-heavy alcohol drinkers. The mean values of non-heavy drinkers were 1.84 ± 0.46, 2.31 ± 0.50 (*p* = 0.04), respectively. The mean values of EUS-SWM were significantly higher in heavy drinkers of more than 60 g of alcohol per day (Table 4).

### 3.4. Receiver Operating Characteristic (ROC) Curve Analysis

**Figure 3:**
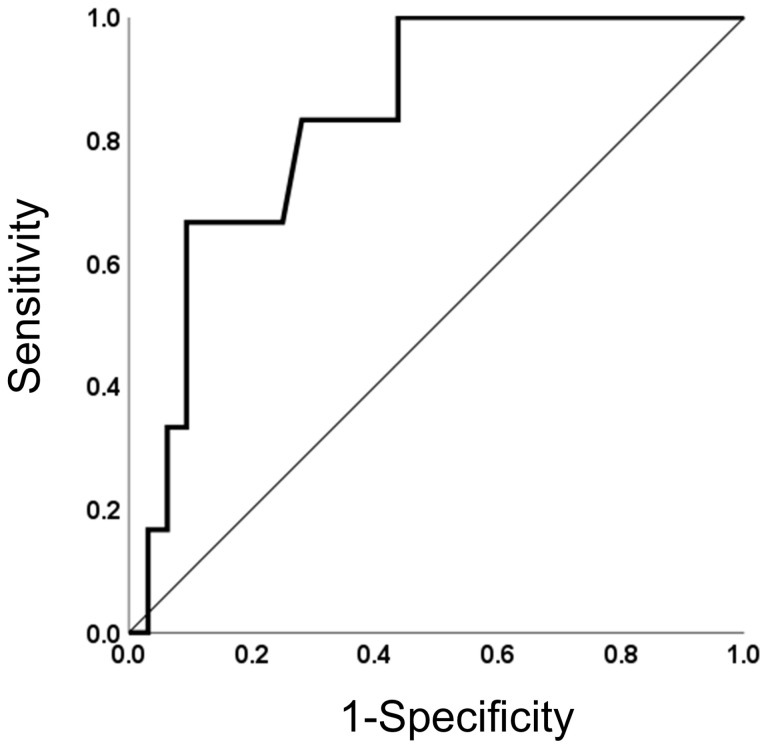
ROC analysis of EUS-SWM.

The area under the ROC for the accuracy of EUS-SWM for the eCP finding group was 0.78 (Figure 3). The cut-off value of 1.87 yielded a sensitivity of 90.9% and a specificity of 63.0%. Positive predictive value and negative predictive value were 50.0% and 94.4% (Table 5).

Receiver operating characteristic curve of endoscopic ultrasonography shear wave measurement for diagnosing eCP.

## 4. Discussion

This study demonstrated the usefulness of EUS-SWM for detecting eCP findings. The diagnosis of eCP is important to prevent progression to establish CP by advising patients that include prohibition, a low-fat diet, and no smoking. In the clinical management of CP, and especially of eCP, disease progression is usually recognized only after substantial structural and functional damage has already occurred, which makes timely therapeutic intervention challenging. The major reason for this difficulty is the lack of reliable biomarkers reflecting disease progression. Although EUS-SWM is relatively more invasive than abdominal ultrasound, CT, or MRI, it may represent a unique and promising tool for the early detection of pancreatic fibrosis or deterioration.

Although diagnostic criteria for eCP have been published by the JPS, the diagnosis of eCP as the criteria are based on EUS findings. Nevertheless, only a few studies have compared EUS findings of eCP with histopathological results, and the eCP diagnostic criteria remain controversial [6]. Moreover, EUS findings of eCP have insufficient interobserver agreement [8,9,19]. In our data, the interobserver agreement rate was also insufficient; the JPS has revised the endoscopic findings of eCP to increase the interobserver agreement rate, but this may still not be sufficient. Therefore, biomarkers and tools were needed to increase the diagnostic capability of eCP. Recently, EUS-SWM has been reported to be useful in the diagnosis of CP [13]. We found that EUS-SWM tended to be significantly higher in the eCP finding group. Our results not only demonstrate the usefulness of EUS-SWM for the diagnosis of eCP but also suggest that eCP finding is associated with increased pancreatic hardness. Our data are also in agreement with previous reports on Rosemont criteria [20], which may help in the diagnosis of eCP, where interobserver agreement is unstable. We found a correlation between eCP findings and EUS-SWM Vs values. The present findings are in concordance with prior reports [18] demonstrating a correlation between eCP findings and EUS-SWM, thereby lending further support to the validity of the underlying hypothesis. EUS-SWM values have also been correlated with the Rosemont classification [15]. CP is currently classified based on mechanistic definitions, but the staging of the disease is not defined; it is conceptual. The definition of eCP in particular remains controversial. However, our results demonstrate that EUS findings of eCP are associated with pancreatic stiffness, proving the correctness of these concepts.

We found that pancreatic hardness was elevated even in cases with only EUS findings of eCP without diagnostic criteria of eCP. Ito et al. reported [21] that some patients who have no symptoms, no past history of acute pancreatitis, and no abnormal gene have EUS findings of eCP. Considering these points, our result indicates that fibrosis of the pancreas may be progressing even in asymptomatic cases, and that even asymptomatic cases may progress to CP. Moreover, EUS-SWM correlates with pancreatic exocrine function [20]. EUS-SWM may be useful for subdividing the stage of eCP.

EUS-SWM is also significantly higher in heavy alcohol drinkers. Although alcohol is known to be a cause of CP and the cause of eCP progression into established CP, it was not known that pancreatic stiffness is increased even before matching the criteria of eCP. Our result may indicate that fibrosis of the pancreas due to heavy alcohol drinking may have begun before the EUS findings. Therefore, measuring pancreatic hardness may help diagnose a pre-eCP. Although studies are needed to increase the number of samples further, these showed that EUS-SWM may be useful as a biomarker other than imaging findings. EUS-SWM is equipped not only on the ARIEETA850 (Fuji Film healthcare, Ltd., Tokyo, Japan) used in this study but also on the ME-3 (OLYMPUS, Tokyo, Japan) and could become a standard feature. If EUS endoscopists around the world perform examinations using EUS-SWM based on this pilot study data, it may be useful in clarifying the pathogenesis of early-stage chronic pancreatitis to chronic pancreatitis.

## 5. Limitations

Our study had several limitations. First, this study was a single-center retrospective study and the sample size was small. There may be some residual confounding due to the small sample size. Second, the interobserver reliability of EUS findings is poor. Dilated side branches are particularly difficult to assess with still images, and interobserver reliability may not increase without video. Third, there is a lack of data on smoking history and body mass index in this study. Despite these limitations, there are several important strengths of this study. Our data complement the hypothesis that eCP findings correlate with pancreatic hardness, suggesting that heavy drinkers may have higher pancreatic hardness even before eCP findings appear. In addition, the procedure for EUS-SWM has not yet been standardized. We measured each case ten times and used the mean value, whereas some reports have used five measurements [18]. Furthermore, the results may be influenced by factors such as visceral fat, which remains an unresolved issue.

## 6. Conclusions

This study showed a correlation between EUS-SWM values and EUS findings of eCP. This suggested that EUS-SWM could be the objective marker of eCP progression. We also found that pancreatic hardness was elevated in heavy alcohol drinkers who did not meet eCP criteria; the results indicated that alcohol was damaging the pancreas before it could be diagnosed by EUS. Although further future research, especially a multicenter prospective study, is needed, EUS-SWM may be used as a less invasive, quantitative biomarker to diagnose, stage, and determine the efficacy of treatment for eCP.

## Figures and Tables

**Table 1:** The patient characteristics.

	Patients Underwent EUS-SWM (N = 38)
Age, years old (range)	68.5 (57.75–75.75)
Male, n (%)	20 (52.6)
Alcohol (≧60 g/day), n (%)	10 (26.3)
Diabetes mellitus, n (%)	5 (13.1)
Chronic pancreatitis, n (%)	10 (26.3)
Autoimmune pancreatitis, n (%)	1 (2.6)
History of acute pancreatitis, n (%)	2 (5.3)
Pancreatic cyst, n (%)	13 (34.2)
Pancreatic screening, n (%)	6 (15.8)
Biliary screening, n (%)	6 (15.8)

EUS-SWM: Endoscopic ultrasonography—shear wave measurement.

**Table 2:** Kappa value of EUS findings.

EUS findings	Kappa value (95% CIs)
Hyperechoic foci; non-shadowing/stranding	0.69 (0.46–0.91)
Lobularity	0.48 (−0.12–1.08)
Hyperechoic MPD margins	0.43 (0.09–0.78)
Dilated side branches	−0.04 (−0.09–0.01)

Cis: Confidence intervals; MPD: main pancreatic duct.

**Table 3:** Comparison between EUS findings and EUS-SWM.

	EUS Findings of e CP ≧ 2 (n = 11)	EUS Findings of e CP < 2 (n = 27)	*p*-Value
Age, years old (range)	60.0 (57.0–75.0)	70.0 (58.0–79.0)	0.49
Male, n (%)	10 (90.9)	10 (37.0)	0.003
EUS-SWM (Vs: m/s)	2.39 ± 0.54	1.84 ± 0.46	0.0065
Chronic pancreatitis, n (%)	6 (54.5)	4 (14.8)	0.02

EUS-SWM: Endoscopic ultrasonography—shear wave measurement; e CP: early chronic pancreatitis.

**Table 4:** Comparison of heavy alcohol drinkers and EUS-SWM.

	Heavy Alcohol Drinker (n = 10)	Non-Heavy Alcohol Drinker (n = 28)	*p*-Value
Age, years old (range)	71.5 (56.75–75.75)	65.0 (57.25–75.25)	0.55
Male (%)	6 (60)	14 (50)	0.43
EUS-SWM (Vs: m/s)	2.31 ± 0.50	1.84 ± 0.46	0.04
Chronic pancreatitis (%)	7 (70.0)	3 (11.1)	0.0009
EUS findings of e CP ≧ 2 (%)	5 (50)	6 (21.4)	0.10

EUS-SWM: Endoscopic ultrasonography—shear wave measurement; eCP: early chronic pancreatitis.

**Table 5:** ROC analysis.

Cut off Value, (m/s)	AUROC (95% CIs)	Sensitivity (%)	Specificity (%)	PPV (%)	NPV (%)
1.87	0.78 (0.62–0.95)	90.9	63.0	50.0	94.4

ROC: Receiver operating curve; CIs: confidence intervals; PPV: positive predictive value; NPV: negative predictive value.

## Data Availability

The original contributions presented in this study are included in the article. Further inquiries can be directed to the corresponding author.

## Abbreviations

The following abbreviations are used in this manuscript:

CP	Chronic pancreatitis
EUS	Endoscopic ultrasonography
EUS-SWM	EUS shear wave measurement
JPS	Japan Pancreas Society
MPD	Main pancreatic duct
ROI	Region of interest
ROC	Receiver operating characteristic
AUROCs	Area under the ROCs
CIs	Confidence intervals
